# Transgenic Mouse Model of Congenital Choledochal Cyst

**DOI:** 10.21203/rs.3.rs-7713827/v1

**Published:** 2025-10-12

**Authors:** Hannah Nicole Rinehardt, Alexis Martyn, Alexander Kolodychak, Masahiro Takeda, Madison Thomas, Lydia Liszewski, Abigail Rutkowski, Alexander Kreger, George Kingsley Gittes

**Affiliations:** Children’s Hospital of Pittsburgh; Children’s Hospital of Pittsburgh; Children’s Hospital of Pittsburgh; Children’s Hospital of Pittsburgh; Children’s Hospital of Pittsburgh; Children’s Hospital of Pittsburgh; Children’s Hospital of Pittsburgh; Children’s Hospital of Pittsburgh; Children’s Hospital of Pittsburgh

**Keywords:** choledochal cyst, genetic mouse model, transgenic, cholestasis, congenital

## Abstract

**Purpose:**

Choledochal cyst is a rare, congenital dilation of the hepatobiliary tree. Due to the associated malignancy risk, complete resection is recommended. There remains a risk of metachronous cholangiocarcinoma despite resection necessitating lifelong surveillance. Choledochal cysts are increasingly prevalent with an incompletely understood connection to carcinogenesis. We sought to develop a mouse model to reliably mimic human disease process of choledochal cyst.

**Methods:**

Experimental transgenic mice were bred with a genotype of Pdx-Cre, TGFα, LSL-KRAS G12D Mu/Wt. Control C57 mice were used as a comparison. Experimental and control mice underwent serial abdominal magnetic resonance imaging (MRI) from weaning to sacrifice.

**Results:**

All experimental mice developed fusiform, extrahepatic common bile duct dilation mimicking a Type I choledochal cyst. Choledochal cyst was present on imaging modalities upon weaning. Maximum common bile duct (CBD) diameter by MRI demonstrated a significantly larger diameter in the experimental group compared to the control group at 10 weeks.

**Conclusion:**

All experimental mice with a genotype of Pdx-Cre, TGFα, LSL-KRAS Mu/Wt developed a phenotype consistent with congenital choledochal cyst. This transgenic mouse model mimics the oncogenic nature of choledochal cyst and could be used to further study disease pathophysiology and novel interventions.

## Introduction

Choledochal cyst or choledochal malformation is a congenital dilation of the intra- or extrahepatic bile ducts with an incidence as high as 1:1000 in East Asian populations [1]. Prior to the 1980s, choledochal cysts were treated with cystenterostomy. In part due to high rates of bile duct carcinoma after cystenterostomy and improved surgical techniques, complete resection became the new standard of care [2]. Despite complete resection and modern surgical methods, there remains a 2% risk of metachronous cholangiocarcinoma at a median of 92 months post-operatively after resection for choledochal malformation [3]. There are case reports of pancreatic head carcinomas occurring after choledochal cyst resection, suggesting a carcinogenic process extending to the proximal pancreatic ducts [4]. Choledochal cysts are increasingly prevalent, yet the etiology and relationship with carcinogenesis are still debated [5, 6]. Anomalous pancreaticobiliary ductal union (APBDU) is highly associated with the disease, and Babbitt’s hypothesis theorizes that reflux of pancreatic enzymes into the bile duct leads to weakening of the ductal wall and subsequent cystic dilation [6, 7]. APBDU without bile duct dilation is also independently associated with a risk of malignancy [7]. Babbitt’s hypothesis has failed to fully explain the evolution of choledochal cysts, and not all patients with APBDU develop biliary dilation [6, 7].

Prior animal models of choledochal malformation are limited to a rat bile duct partial ligation model and a canine APBDU model, both of which lead to reliable cystic dilation of the extrahepatic bile duct [8, 9]. The canine model is technically difficult, involving the anastomosis of the bile duct to the pancreatic duct in the pancreatic head, and this model also necessitates expensive and time-consuming large animal surgical and post-operative care [9, 10]. The disease model in rats involves a partial obstruction mechanism, which could replicate the Sphincter of Oddi dysfunction that is often seen in patients with choledochal cysts [8, 11]. Previous animal models have not replicated the congenital nature of human disease and require surgical interventions which can be variable and present a confounding factor to studying the disease process. A genetic mouse model could facilitate the study of choledochal cyst pathogenesis and avoid the confounding aspects of prior models.

We sought to develop a mouse model of clinical choledochal malformation to aid in further investigations of the disease etiology and connection with hepatobiliary malignancy risk. Our model involves breeding mice with pancreas-specific KRAS G12D oncogenic mutation plus overexpression of TGF alpha that is regulated by the metallothionein 1 promoter in the liver and pancreas [12, 13]. These oncogenes are associated with cystic ductal tumor development in prior mouse models [12–15]. The pancreatic aspect of oncogene expression is pursued in this model based on known intrapancreatic portions of choledochal cysts and the shared common endodermal embryologic origin of the distal bile duct and proximal pancreatic duct [4]. Given the strong association between choledochal cyst and APBDU, plus the location of the duct junction within the pancreatic head, we hypothesize that targeting the hepatic and pancreatic ducts in the genetic model early on in embryonic development could lead to choledochal cyst in experimental mice. A reliable choledochal cyst mouse model should lead to cystic dilation of the extra- and/or intrahepatic bile ducts without evidence of biliary obstruction.

## Materials and methods

### Mouse breeding

All mouse handling and experimental procedures were approved by the Animal Research and Care Committee at the Children’s Hospital of Pittsburgh and the University of Pittsburgh Institutional Animal Care and Use Committee. Mouse colonies were maintained in a specific pathogen-free barrier facility in the Children’s Hospital of Pittsburgh of UPMC. Hemizygous, global TGFalpha(TGFα)-overexpressing mice (Strain No. 003722) were purchased through cryorecovery services from Jackson Laboratories (Jax). This mutation is regulated by the metallothionein 1 promoter in the liver and pancreas. These mice were crossed with Lox-STOP-Lox(LSL)-Kras^G12D^ mice (Jax Strain No. 008179) and Pdx1-Cre mice (Jax Strain No. 014647). Using the Cre-Lox system, the LSL-KRAS oncogene is paired with the pancreas-specific promoter Pdx1-Cre. This results in tissue-specific expression of the heterozygous LSL-Kras^G12D^ mutation in the pancreas. These mouse strains were bred to generate experimental mice with the genotype TGFα; Pdx1-Cre; LSL-Kras^G12D^-Mu/Wt. Mouse genotypes were confirmed by PCR of tail biopsies using Jax-recommended primers.

### Cholangiogram

One control and one experimental mouse underwent computed tomography (CT) cholangiography at 12 weeks old. Under isoflurane anesthesia, a midline laparotomy was performed, and the duodenum was retracted caudally. Needle duodenotomy was performed opposite the ampulla using a 27 G needle. A 28 G catheter was inserted into the duodenotomy and through the ampulla. The catheter was clamped near the insertion site. A viscous contrast solution of 10% barium sulfate (Thermo Fisher Scientific) and 0.1% gelatin (Millipore Sigma) was infused at a flow rate of 50 μL/min. Total infusion volume was dependent on body weight (1.2 μL/g). After removing the catheter, the duct was immediately tied to prevent backflow and radiographic artifact. Mice were sacrificed, and livers were harvested *en bloc* with the duodenum and pancreatic head for immediate CT imaging.

Micro-CT imaging was acquired using a Siemens Inveon Multi-Modality microPET/SPECT/CT system (Siemens Medical Solutions USA, Inc., Knoxville, TN 37932) at 80 kV and 500 μA, with an exposure of 650 ms. A transaxial field of view (FOV) of 27.01 mm and an axial FOV of 35.04 mm with 2×2 binning at high magnification offered a resolution of 22.81 μm. An aluminum filter was used to minimize beam hardening. A total of 360 projections were collected over a 360° rotation, with a rotation step size of 1° and a settle time of 1000 ms per projection. Image reconstruction was performed using a Feldkamp algorithm, with no downsampling, application of a Shepp-Logan filter, and slight noise reduction. Beam-hardening correction was not applied. The images underwent Hounsfield Unit calibration to accurately characterize tissue radiodensity. 3D maximum intensity projection videos were created using Inveon Research Workplace (IRW version 2.1). Cholangiograms were analyzed using RadiAnt DICOM Viewer (Medixant).

### Magnetic Resonance Imaging

T2-Weighted Abdominal MRI images were obtained upon weaning at 4–6 weeks old and again at 8–10 weeks old of experimental mice (n = 5) under isoflurane anesthesia. Images were obtained from control C57 mice (n = 3) at 10 weeks old. Images were evaluated using ImageJ, Java 1.8.0 (National Institutes of Health, USA). Maximum bile duct diameter was measured using coronal images in the T2 phase in ImageJ to identify the point of maximal dilation. Images were obtained using a Bruker 7T BioSpec 70/30 USR spectrometer (Bruker BioSpin MRI, Billerica, MA). A 35-mm quadrature volume coil was used for both transmission and reception.

### Ultrasound Imaging

Abdominal ultrasounds were performed on experimental mice (n = 5) upon weaning at 4–6 weeks old and again at 8–10 weeks old under isoflurane anesthesia. Linear probe was utilized in the transverse position, and images were evaluated in ImageJ, Java 1.8.0 (National Institutes of Health, USA). Maximum bile duct diameter was measured by scanning through the length of the bile duct from the hilum of the liver to the ductal bifurcation down to the pancreatic head and finding the point of maximal dilation. VisualSonics Vevo 770^®^ High-Resolution Imaging System was used for obtaining these images (FUJIFILM VisualSonics, Inc., Toronto, ON, Canada).

### Serum analysis

Weekly blood collections were carried out on experimental and control mice from weaning to sacrifice (4–12 weeks old). Microvette CB 300 Capillary Blood Collection Tubes were used to collect approximately 200 μL of tail vein blood, which was centrifuged for 15 minutes at 4°C. Aliquoted serum samples were stored at −20°C until testing the following biochemical parameters: alanine aminotransferase (ALT), aspartate aminotransferase (AST), total bilirubin, and γ-glutamyl transferase (GGT). Serum transaminase concentrations were measured by ELISA (Abcam; ALT: ab282882, AST: ab263882). Total bilirubin and GGT were quantified using colorimetric assays (Abcam, ab235627; Elabscience, E-BC-K126-M). All tests were conducted according to manual instructions.

### Intraperitoneal glucose tolerance test

10-week-old mice were fasted overnight for 16 hours and injected intraperitoneally with 2 g/kg D-glucose solution (Sigma-Aldrich). A glucometer (Contour NEXT EZ) was used to measure serial capillary blood glucose levels from the tail vein at 0, 15, 30, 60, 90, and 120 minutes post injection.

### Tissue processing and histology

Harvested common bile ducts and livers were fixed in 4% paraformaldehyde (PFA) for 48 hours at 4°C then transferred into 30% sucrose for an additional 48 hours. Cryoprotected tissues were embedded in Tissue-Tek OCT, frozen via dry ice, and stored at −20°C for one hour prior to cutting 5μm sections. H&E and Masson’s Trichrome staining were performed for histological assessment. Additionally, harvested pancreata were fixed in 4% PFA overnight at 4°C and prepared into 5 μL paraffin-embedded sections at the CHP Rangos histology core facility followed by H&E staining. All brightfield images were acquired using the Leica Aperio CS2 slide scanner at 20x and 40x magnification.

For immunohistochemistry, frozen CBD and liver sections were rehydrated in PBS for 10 minutes prior to performing antigen retrieval using heat and citrate buffer (pH 6.0). Slides were then incubated overnight at 4°C with rabbit anti-CK19 (1:200, Abcam, ab133496) primary antibody after serum blocking for 1 hour at room temperature. The next day, slides were incubated with fluorescent-conjugated (CY5) secondary antibody (1:300, Jackson ImmunoResearch Labs) for 1 hour at room temperature. Fluorescence images were captured using the Leica DMi8 (2x and 10x magnification) with Leica Application Suite X (LAS X).

### Statistical Analysis

Kaplan-Meier methods were used to plot survival curves and were analyzed by using log-rank analysis tests. Survival analyses were completed using Stata Standard Edition, version 18.5 (College Station, TX, USA). Continuous variables were graphed and compared using unpaired student’s t-test in GraphPad Prism, version 10.0.0 (Boston, MA, USA). P values ≤ 0.05 were considered significant. For glucose tolerance testing, multiple unpaired t-tests were utilized. For imaging modalities comparison, multiple one-way ANOVA was performed.

## Results

**Figure 1:**
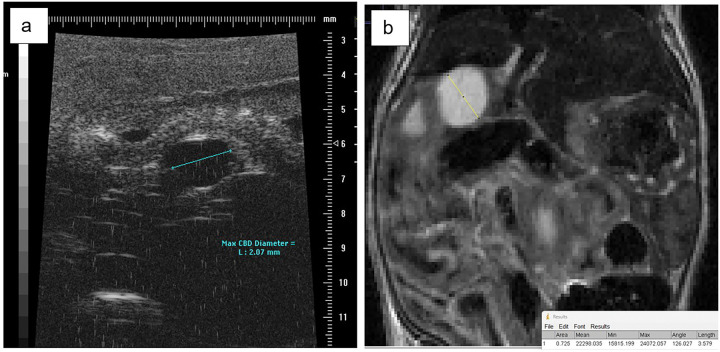
**a** Measurement of the maximum bile duct diameter of an experimental mouse at 4 weeks of age by abdominal ultrasonography (length = 2.07 mm) **b** Measurement of the maximum bile duct diameter of an experimental mouse at 5 weeks of age by coronal T2-weighted abdominal MRI (length = 3.579 mm)

**Figure 2:**
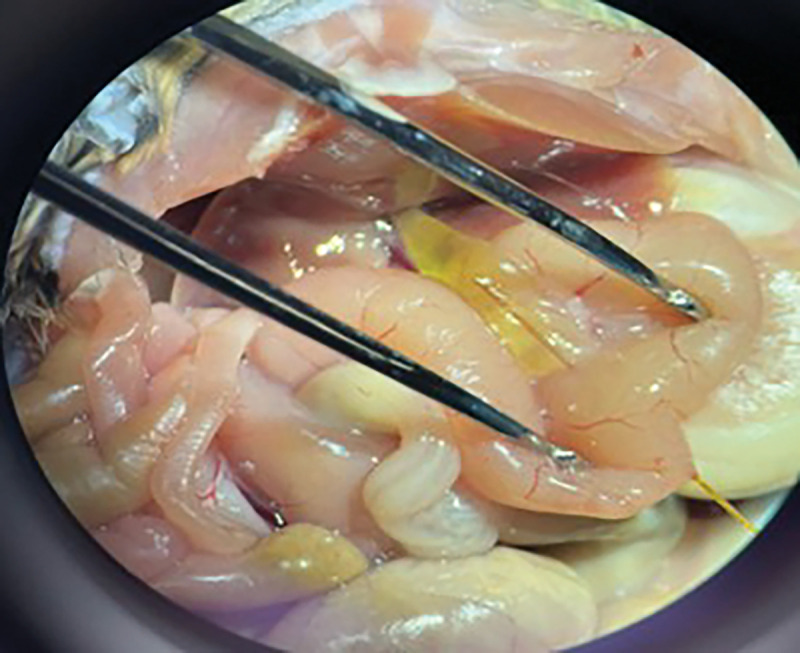
Blunt tip catheter inserted through the ampulla in a transduodenal fashion at necropsy at 12 weeks of age in an experimental mouse easily passes into the cyst lumen (Catheter tip within the cyst lumen denoted by green arrowhead)

**Figure 3:**
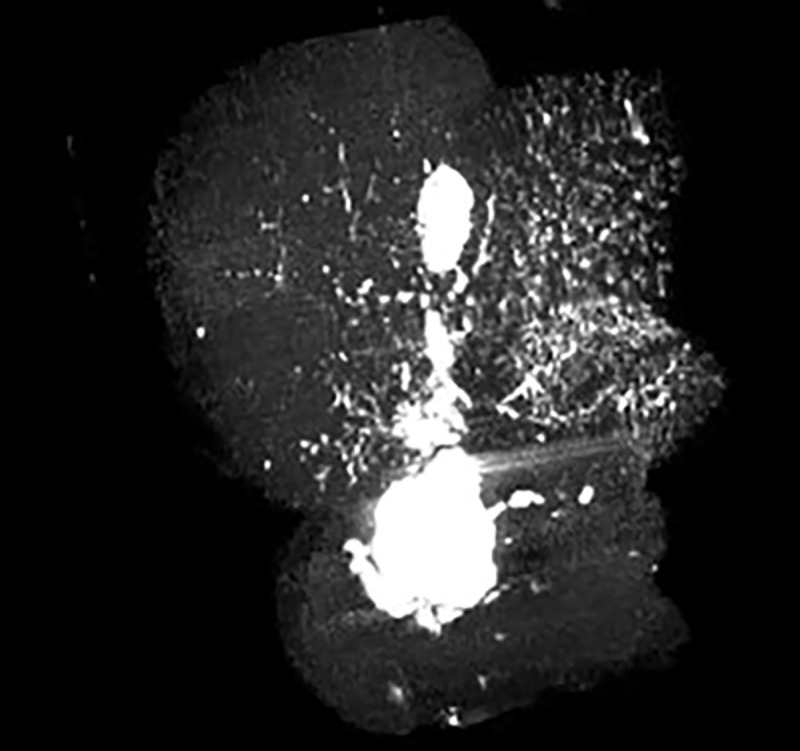
Experimental mouse at 12 weeks of age demonstrates fusiform extrahepatic bile duct dilation (green arrowhead) on CT cholangiogram (the gallbladder is denoted by the green arrow)

mouse at 5 weeks demonstrates the appearance of congenital cystic extrahepatic duct dilation (Fig. 1b). Mice were sacrificed at 12 weeks of age and cysts were measured using a ruler under a dissection microscope to obtain gross maximum extrahepatic cyst diameter at necropsy. At necropsy, a blunt tip catheter was inserted into the ampulla in a trans-duodenal fashion to evaluate for evidence of biliary obstruction (Fig. 2). The catheter easily passes into the cyst lumen demonstrating lack of distal ductal obstruction as an etiology for cyst development (n = 5). In one control mouse and one experimental mouse, a cholangiogram was performed at 12 weeks of age. This was done to evaluate the morphology of intra and extrahepatic bile ducts and again evaluate for evidence of biliary obstruction (Fig. 3). This demonstrated fusiform cystic dilation of the extrahepatic bile duct and non-dilated, non-cystic intra-hepatic bile ducts. There was no evidence of biliary obstruction on CT cholangiography. This appearance is consistent with the phenotype of type I choledochal cysts in humans [16].

**Figure 4:**
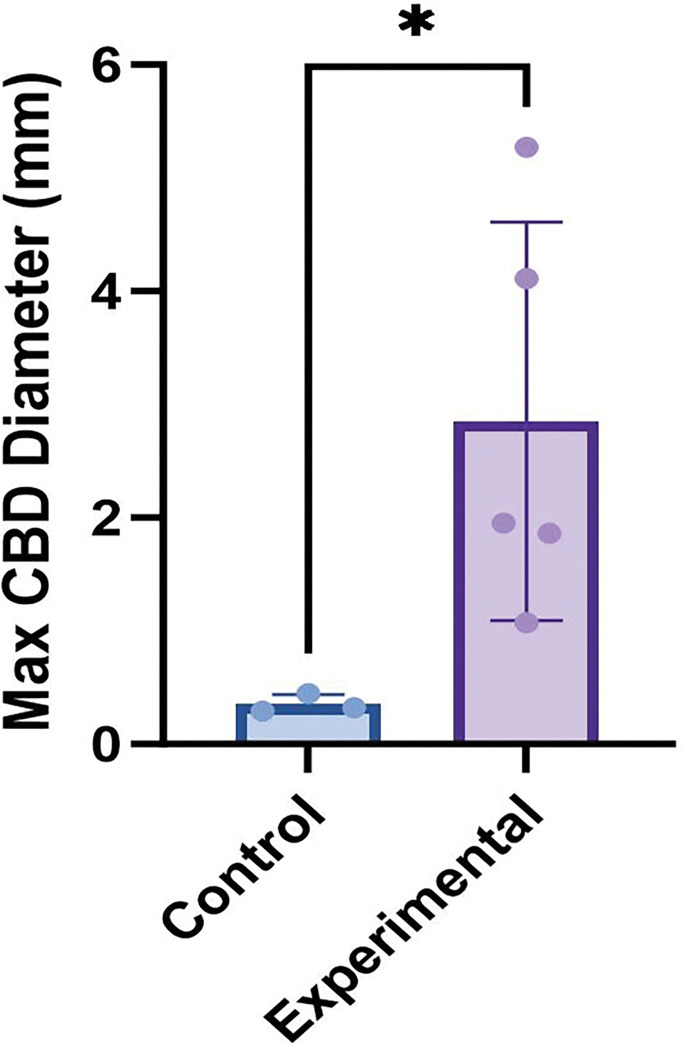
Maximum bile duct diameter of experimental and control mice at 10 weeks of age measured by coronal MRI (p-value = 0.036)

**Figure 5:**
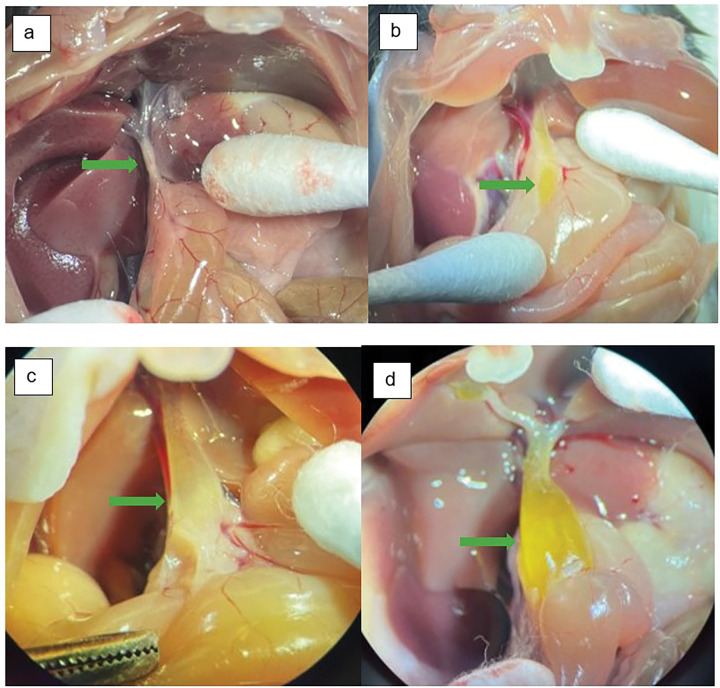
Mouse bile ducts were photographed upon necropsy at 12-weeks of age; The green arrow in each image denotes the point of approximate maximum extrahepatic bile duct diameter **a** control mouse **b**experimental mouse with mild choledochal cyst **c** experimental mouse with moderate choledochal cyst **d** experimental mouse with severe choledochal cyst

Given that the in situ choledochal malformation could be quite tortuous, the coronal view of the T2-weighted MR abdomen was suspected to be the most accurate way to identify and measure the maximum bile duct diameter. In five experimental mice, maximum bile duct diameters were compared on 10-week ultrasound, 10-week axial MRI view, 10-week coronal MRI view, and 12-week necropsy (considered the gold standard). The results are summarized in Supplemental Fig. 1, with the coronal view MRI mean duct diameter being the closest to necropsy measurement mean by multiple one-way ANOVA (p-value = 0.9159). The coronal view T2-MRI bile duct diameters were then compared between control (n = 3) and experimental (n = 5) mice by unpaired t-test (Fig. 4) with a significantly dilated bile duct diameter in experimental mice compared to controls (p-value = 0.036). Given the heterogeneity of the degree of biliary dilation between experimental mice (Fig. 5a–d), a severity scale was created (Table 1) with a diameter < 0.50 mm considered normal (n = 3), maximum diameter 0.50–1.5 mm considered mild dilation (n = 3), 1.5–3.0 mm considered moderate dilation (n = 3), and a maximum diameter > 3.0 mm severe dilation (n = 4). This scale is based on the distributions of data noted in the cohorts of experimental and normal mice. Maximum diameter by T2-weighted MRI were compared at the early and late timepoints, and there was no evidence of progressive dilation or cyst growth over time in 5 mice with a p-value of 0.112 (Supplemental Fig. 2).

**Figure 6:**
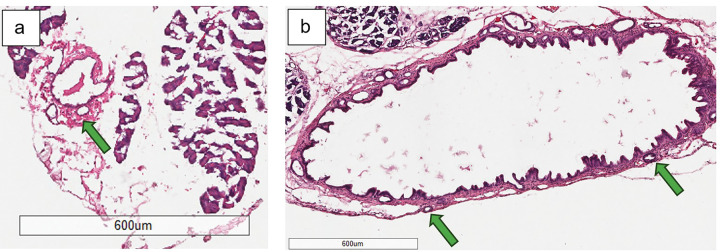
**a** Hematoxylin and eosin staining of control mouse bile duct harvested at 12-weeks of age (4x magnification); normal intramural peribiliary gland denoted by green arrow **b** Hematoxylin and eosin staining of experimental mouse extrahepatic bile duct harvested at 12-weeks of age (4x magnification); normal intramural peribiliary glands denoted by green arrows

**Figure 7:**
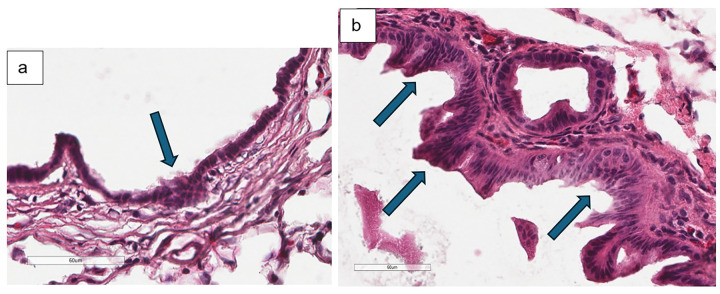
**a** Hematoxylin and eosin staining of experimental mouse bile duct harvested at 12-weeks old demonstrates minimal epithelial changes as illustrated by focal hyperplasia indicated by blue arrow (40x magnification) **b** another experimental mouse exhibits mild epithelial changes as illustrated by diffuse hyperplasia indicated by blue arrows (40x magnification)

At 12-week necropsy, the liver, pancreas, and bile duct were harvested separately for histologic analysis. There was no evidence of hepatic biliary congestion on harvesting of the liver (Figure 5b–d). Hematoxylin and eosin staining demonstrates a normal bile duct in control mice with peribiliary glands (Figure 6a). In the experimental mice, a massively dilated bile duct is present again with normal intramural and extramural peribiliary glands (Figure 6b). The H&E slides were scored using the semi-quantitative epithelial lining and mural score (ELMS) [6]. Experimental mice bile duct specimens were consistent with minimal and mild epithelial changes of choledochal cyst (Figure 7a–b). Cytokeratin-19 staining of control and experimental mice reveals preserved staining of the bile duct epithelium in both groups demonstrating a maintenance of bile duct epithelium differentiation in mice with choledochal malformation (Supplemental Figure 3). Masson’s trichrome staining of the liver and bile ducts in both groups was performed. The presence of normal collagen in the wall of the extrahepatic bile duct was noted in both groups and served as a positive control. Absent staining for Masson’s trichrome in the liver suggests the absence of liver fibrosis (Supplemental Fig. 4a-b). H&E staining of the pancreas in experimental mice demonstrates normal acinar tissue, normal islets and scattered pancreatic intraepithelial neoplasms (Supplemental Fig. 5). No evidence of invasive carcinoma nor large cystic lesions were identified over the 12-week course.

**Figure 8:**
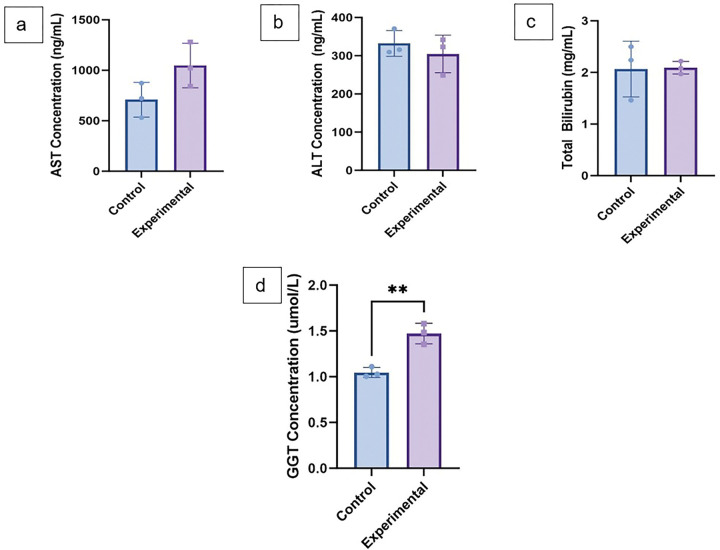
**a** Serum collected from control and experimental mice at 7–11 weeks of age **a**serum AST (p-value 0.104) **b** serum ALT (p-value 0.470) **c** serum total bilirubin (p-value = 0.941) **d** serum GGT (p-value 0.0042); Unpaired student’s t-test (** denotes p-value <0.01).

Serum Alanine Aminotransferase (ALT), Aspartate Aminotransferase (AST) and total bilirubin were compared between groups and were not significantly different between groups. Gamma-glutamyl transferase (GGT) levels were higher in experimental mice (p = 0.0042) suggesting subclinical cholestasis given the lack of jaundice in the mice (Fig. 8a–d). Intraperitoneal glucose tolerance testing was performed at 10 weeks of age with normal glucose homeostasis in both groups (Supplemental Fig. 6). Experimental mice had supranormal glucose tolerance at fasting and at 2 minutes. Experimental mice were not jaundiced; two mice had decreased activity and abdominal distension at 10–12 weeks old and were sacrificed. Survival during the study period was not statistically different between mice by log-rank analysis with a p-value of 0.224 (Supplemental Fig. 7). Body weight between groups was not different (Supplemental Fig. 8) at 6 weeks or 12 weeks old (p-values 0.607 and 0.107 respectively). The experimental mice had a higher body weight at 10 weeks old compared to control mice (p = 0.0042).

## Discussion

Pancreas-specific KRAS-G12D mutation and TGF alpha overexpression in the liver and pancreas have been separately associated with cystic tumors in previous mouse models [12–15]. Our data demonstrate that when combined, the chosen oncogenic mutations lead to a mouse model consistent with Type 1 choledochal cyst. Type 1 choledochal cyst is the most common form of choledochal malformation and has the highest association with metachronous biliary carcinoma [3, 16]. Despite complete resection of choledochal cysts, there is a risk of later development of cholangiocarcinoma, gallbladder carcinoma, or pancreatic head carcinoma [3, 4]. Serum liver function testing and serum cancer antigen 19–9 plus abdominal ultrasounds are recommended after resection for lifelong malignancy surveillance in these patients [3]. Prior animal models have replicated APBDU or Sphincter of Oddi dysfunction that led to bile duct dilation in canines and rats but have not connected cyst development with malignancy risk [8–10]. We present a mouse model which replicates the congenital nature of disease and genetic oncogenic risk by a different mechanism than prior models.

In the present disease framework, the choledochal cyst is present at weaning and is non-progressive over 2–3 months. Compared with control mice, the extrahepatic bile duct is significantly dilated without evidence of biliary obstruction. Serum testing reveals similar transaminase levels and total bilirubin compared to normal mice. Subclinical cholestasis is suggested by elevated serum GGT in experimental mice. Histologically, the bile ducts of experimental mice are consistent with human choledochal cyst specimens with focal and diffuse hyperplasia present in the bile duct epithelium [6]. Like the previous rodent model, a mouse-specific severity scale of choledochal cyst was developed (Table 1)[8]. From a logistical perspective, mouse breeding is relatively straightforward, no surgical intervention is required to induce the disease phenotype, and most mice survived until the 12-week endpoint without signs of distress.

An unexpected histologic finding is the presence of intraepithelial neoplasms in the pancreas (PanINs) of experimental mice (Supplemental Fig. 5). These do not cause any detectable clinical signs over the 3-month course of the study but could theoretically progress to more invasive tumors if a longer experiment were pursued in these mice. The glucose tolerance was preserved despite the presence of PanINs at 10 weeks old, suggesting these represent an incidental finding (Supplemental Fig. 6). A limitation of our study is the induced singular genetic cause leading to choledochal cyst whereas human disease is more heterogeneous in nature with multiple genetic and environmental risk factors impacting disease development, progression and lifetime malignancy risk [3, 5, 6]. We compared the findings to clinical choledochal cyst literature but did not directly compare our tissues to any human samples. We only studied our mice out to 12 weeks given that we aimed to assess the ease of use of this mouse model at early timepoints for feasible experimentation. We did not evaluate long-term survival or hepatopancreaticobiliary malignancy rates in the model, although this may be relevant in other studies and is unanswered by our investigation.

## Conclusion

We demonstrate a feasible genetic mouse model of congenital type I choledochal cyst. Our data suggest that oncogenes could be involved not only as a downstream result of pancreaticobiliary reflux but could also be implicated in the development of the disease. This model can be used in future studies to further investigate oncogenic aspects of the disease process and as a platform to test relevant therapeutics in vivo.

## Supplementary Material

Supplementary Files

This is a list of supplementary files associated with this preprint. Click to download.

• SupplementalFiguresCCPSI.docx

## Figures and Tables

**Table 1:** Choledochal cyst severity scale based on the distribution of maximum bile duct diameter as measured on coronal T2-weighted abdominal MRI

	Normal	Mild Dilation	Moderate Dilation	Severe Dilation

Maximum Bile	<0.50	0.5–1.5	1.5–3.0	>3.0
Duct Diameter (mm)				

## Data Availability

Data is provided within the manuscript files. Any additional data or analysis are available from the corresponding author on reasonable request.

## References

[1] Yamaguchi M (1980) Congenital choledochal cyst. Analysis of 1,433 patients in the Japanese literature. Am J Surg. ;140(5):653–7. 10.1016/0002-9610(80)90051-3. 6776832

[2] Caudle SO, Dimler M (1986) The current management of choledochal cyst. Am Surg 52(2):76–80 3511809

[3] Koea J, O’Grady M, Agraval J, Srinivasa S (2022) Defining an optimal surveillance strategy for patients following choledochal cyst resection: results of a systematic review. ANZ J Surg 92(6):1356–1364. 10.1111/ans.17775Epub 2022 May 17. 35579057

[4] Schmuck RB, de Carvalho-Fischer CV, Neumann C, Pratschke J, Bahra M (2016) Distal bile duct carcinomas and pancreatic ductal adenocarcinomas: postulating a common tumor entity. Cancer Med 5(1):88–99. 10.1002/cam4.566Epub 2015 Dec 9. 26645826 PMC4708893

[5] Soares KC, Kim Y, Spolverato G, Maithel S, Bauer TW, Marques H, Sobral M, Knoblich M, Tran T, Aldrighetti L, Jabbour N, Poultsides GA, Gamblin TC, Pawlik TM (2015) Presentation and Clinical Outcomes of Choledochal Cysts in Children and Adults: A Multi-institutional Analysis. JAMA Surg. ;150(6):577–84. 10.1001/jamasurg.2015.0226. 25923827

[6] Turowski C, Knisely AS, Davenport M (2011) Role of pressure and pancreatic reflux in the aetiology of choledochal malformation. Br J Surg. ;98(9):1319–26. 10.1002/bjs.7588. Epub 2011 Jul 4. 21725969

[7] Hasumi A, Matsui H, Sugioka A, Uyama I, Komori Y, Fujita J, Aoki H (2000) Precancerous conditions of biliary tract cancer in patients with pancreaticobiliary maljunction: reappraisal of nationwide survey in Japan. J Hepatobiliary Pancreat Surg. ;7(6):551–5. 10.1007/s005340070003. 11180886

[8] Zhang SH, Zhang YB, Cai DT, Pan T, Chen K, Jin Y, Luo WJ, Huang ZW, Chen QJ, Gao ZG (2024) Preliminary exploration of animal models of congenital choledochal cysts. World J Gastroenterol 30(10):1420–1430. 10.3748/wjg.v30.i10.1420 38596496 PMC11000093

[9] Ohkawa H, Sawaguchi S, Yamazaki Y, Sakaniwa M, Ishikawa A (1981) The production of anomalous pancreaticobiliary ductal union in canine models. Z Kinderchir. ;32(4):328–36. 10.1055/s-2008-1063280. 7282070

[10] Han SJ, Han A, Kim MJ, Kim H The role of sphincteroplasty in adverse effect of anomalous pancreaticobiliary duct union in an animal model. Pediatr Surg Int. 2006 Sept 20;23(3):225–231. doi. 10.1007/s00383-006-1787-4. 17021737

[11] Craig AG, Chen LD, Saccone GT, Chen J, Padbury RT, Toouli J (2001) Sphincter of Oddi dysfunction associated with choledochal cyst. J Gastroenterol Hepatol. ;16(2):230–4. 10.1046/j.1440-1746.2001.02365.x. 11207909

[12] Jackson EL, Willis N, Mercer K, Bronson RT, Crowley D, Montoya R, Jacks T, Tuveson DA (2001) Analysis of lung tumor initiation and progression using conditional expression of oncogenic K-ras. Genes Dev 15(24):3243–3248. 10.1101/gad.943001 11751630 PMC312845

[13] Jhappan C, Stahle C, Harkins RN, Fausto N, Smith GH, Merlino GT (1990) TGF alpha overexpression in transgenic mice induces liver neoplasia and abnormal development of the mammary gland and pancreas. Cell. ;61(6):1137–46. 10.1016/0092-8674(90)90076-q. 2350785

[14] Li J, Wei T, Zhang J, Liang T (2021) Intraductal Papillary Mucinous Neoplasms of the Pancreas: A Review of Their Genetic Characteristics and Mouse Models. Cancers (Basel) 13(21):5296. 10.3390/cancers13215296 34771461 PMC8582516

[15] Hingorani SR, Petricoin EF, Maitra A, Rajapakse V, King C, Jacobetz MA, Ross S, Conrads TP, Veenstra TD, Hitt BA, Kawaguchi Y, Johann D, Liotta LA, Crawford HC, Putt ME, Jacks T, Wright CV, Hruban RH, Lowy AM, Tuveson DA (2003) Preinvasive and invasive ductal pancreatic cancer and its early detection in the mouse. Cancer Cell. ;4(6):437–50. 10.1016/s1535-6108(03)00309-x. 14706336

[16] Todani T, Watanabe Y, Narusue M, Tabuchi K, Okajima K (1977) Congenital bile duct cysts: Classification, operative procedures, and review of thirty-seven cases including cancer arising from choledochal cyst. Am J Surg. ;134(2):263–9. 10.1016/0002-9610(77)90359-2. 889044

